# Evaluation of Force Decay in Intraoral Elastics With Different Lumen Sizes: An In Vitro Study

**DOI:** 10.7759/cureus.97809

**Published:** 2025-11-25

**Authors:** Santha Kumari, Sathi Rami Reddy Mora, V Vijaykumar, Sruthi Jeevagan, Parvathy Kurup R, Devadharshini Kirubhaharan

**Affiliations:** 1 Department of Orthodontics and Dentofacial Orthopedics, Adhiparasakthi Dental College and Hospital, Melmaruvathur, IND

**Keywords:** elastic degradation, force decay, intraoral elastics, lumen size, mouthwash exposure

## Abstract

Introduction: Orthodontic elastics are routinely used for molar correction and space closure; however, their clinical efficiency may be compromised by gradual force degradation. Since elastics of different lumen sizes may be prescribed sequentially for the same patient, assessing their stability and the influence of mouthwash exposure is clinically relevant.

Materials and methods: Latex intraoral elastics (OSL Orthodontic Intraoral Elastics) of five different lumen sizes (1/8”, 3/16”, 1/4”, 5/16”, and 3/8”), each designed to deliver 3.5 oz of force, were tested. Fifty samples were divided into two groups: Group A (stored in artificial saliva) and Group B (treated with chlorhexidine mouthwash at predetermined intervals). The elastics were extended to three times their lumen size using custom jigs. Force values were recorded at baseline, one, three, 12, and 24 hours using a digital gauge, and the percentage of force decay was calculated. Data were analyzed using one-way analysis of variance (ANOVA) and independent *t*-tests, with p < 0.05 as the criterion for significance.

Results: All elastics demonstrated progressive force decay over time. Larger lumen elastics (A3 and A4) maintained force more effectively, whereas smaller lumen sizes (A1 and A2) exhibited greater force loss, particularly at 12-24 hours. Mouthwash exposure produced variable effects, with significant differences observed among selected elastics at specific intervals.

Conclusions: Lumen size plays a critical role in the force decay of intraoral elastics, with smaller lumen elastics showing earlier and greater force loss. Mouthwash exposure inconsistently alters this pattern, emphasizing the need for careful patient selection and guidance in clinical practice.

## Introduction

Orthodontic elastics are widely used to achieve tooth movement, such as correcting molar relationships, midline correction, and closing extraction spaces. However, their effectiveness decreases over time due to force degradation, a process influenced by material properties, manufacturing processes, lumen size, and environmental factors [1].

The lumen size of an elastic significantly affects its force retention, with smaller lumen elastics losing force more rapidly than larger lumen ones. This is likely due to increased internal stress and reduced elasticity in smaller diameters, as well as the extent of elastic stretch and the initial force applied. Most force loss occurs within the first 24 hours after stretching, emphasizing the importance of understanding how elastic dimensions influence treatment mechanics [2].

External factors, such as exposure to mouthwashes, can affect the rate at which intraoral elastics lose force. Mouthwashes containing alcohol or chlorhexidine may accelerate force reduction, potentially compromising the effectiveness of elastics [3]. These observations emphasize the importance of selecting appropriate elastics and guiding patients on oral hygiene to ensure stable and effective force delivery during orthodontic treatment.

The purpose of this study is to determine whether force decay varies with different lumen sizes of intraoral elastics when forces are kept constant. Intraoral elastics are available in different dimensions -1/8”, 3/16”, 1/4", 3/8”, 5/16” with lumen sizes of 3.2 mm, 4.8 mm, 6.4 mm, 8.0 mm, 9.5 mm, respectively. These elastics, when stretched three times their lumen size, deliver a light (2.5 oz), medium (3.5 oz), or heavy (4.5 oz) force, depending on the material's thickness [4]. Intraoral elastics are selected for a patient by measuring the distance between the dental units where the elastics need to be engaged, as well as the required force. It may be necessary to use different intraoral elastic sizes (keeping the force constant) for the same patient at different time points due to changes in the distance between the dental units resulting from orthodontic tooth movement. Hence, when delivering different intraoral elastics (keeping the force constant) at different times for the same patient, depending on the distance, it is necessary to determine whether there is a difference in the force decay of each at different intervals during their wear.

The present study is designed to assess the force decay of intraoral elastics with varying lumen sizes and to examine the effect of mouthwash exposure on their force retention, offering practical insights for improving clinical outcomes in orthodontics.

## Materials and methods

A power analysis using the G*Power computer program (Heinrich-Heine-Universität Düsseldorf, Düsseldorf, Germany) indicated that a total sample of 48 would be needed (24 in each group) to detect significant effects (d=0.9) with 85% power by comparison of means between two groups by t-test with alpha at 0.05. Inclusion criteria were intraoral latex elastics of different lumen sizes with the same force specification, elastics within their expiry dates, and elastics stored in sealed plastic packages in a cool, dark environment. The study excluded elastics that were pre-stretched, deformed, or damaged to ensure consistency and reliability of the results.

**Figure 1:**
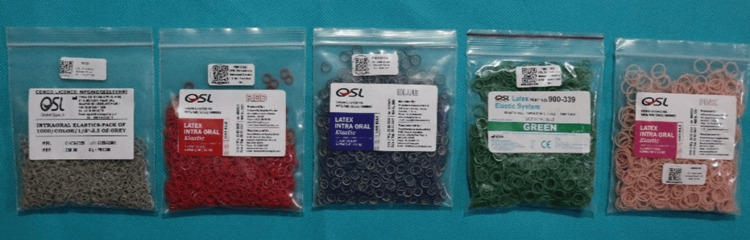
Intraoral latex elastics OSL Orthodontic Intraoral Elastics (grey 1/8”, red 3/16”, blue 1/4”, green 5/16”, pink 3/8”) producing 3.5 oz of force.

In this study, intraoral latex elastics (OSL Orthodontic Intraoral Elastics - grey 1/8”, red 3/16”, blue 1/4”, green 5/16”, and pink 3/8”) producing 3.5 oz of force (Figure 1), within their expiration dates and stored in sealed plastic packages, were used.

**Figure 2:**
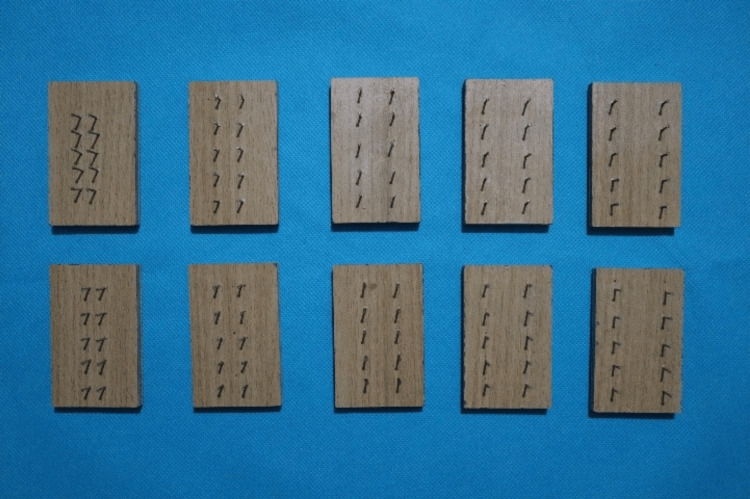
Fabrication of custom jigs The distance between the pins in custom jigs depends on the size of the elastics when stretched three times their lumen size ( 1/8” - 9.5 mm, 3/16” - 14.3 mm, 1/4” - 19.1 mm, 5/16” - 23.8 mm, 3/8” - 28.6 mm).

The study consists of two groups: Group A (elastics stored in artificial saliva without exposure to mouthwash) and Group B (elastics stored in artificial saliva with exposure to chlorhexidine mouthwash at predetermined intervals). Fabrication of custom jigs for both groups [5]. The distance between the pins in custom jigs is measured using a digital vernier caliper and depends on the size of the elastics when stretched three times their lumen size (1/8” - 9.5 mm; 3/16” - 14.3 mm; 1/4” - 19.1 mm; 5/16” - 23.8 mm; 3/8” - 28.6 mm) (Figure 2) [6].

**Figure 3:**
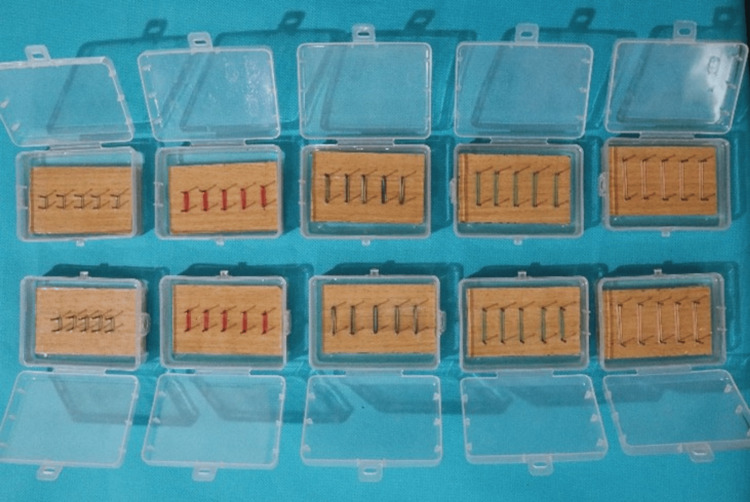
Intraoral elastics of different dimensions (1/8”, 3/16”, 1/4", 5/16”, and 3/8”, each producing 3.5 oz of force ) engaged in five different custom jigs and immersed in artificial saliva

In this in vitro study, intraoral elastics of different dimensions (1/8", 3/16", 1/4", 5/16", and 3/8") in Group A, each producing 3.5 oz of force [1], were engaged in five different custom jigs and immersed in artificial saliva (Figure 3). Force measurements were recorded at multiple time points: baseline, one, three, 12, and 24 hours [7]. In Group B, the elastics were exposed to chlorhexidine mouthwash for 60 seconds three times a day at baseline, 12, and 24 hours to evaluate the effect of mouthwash exposure on force decay [8].

**Figure 4:**
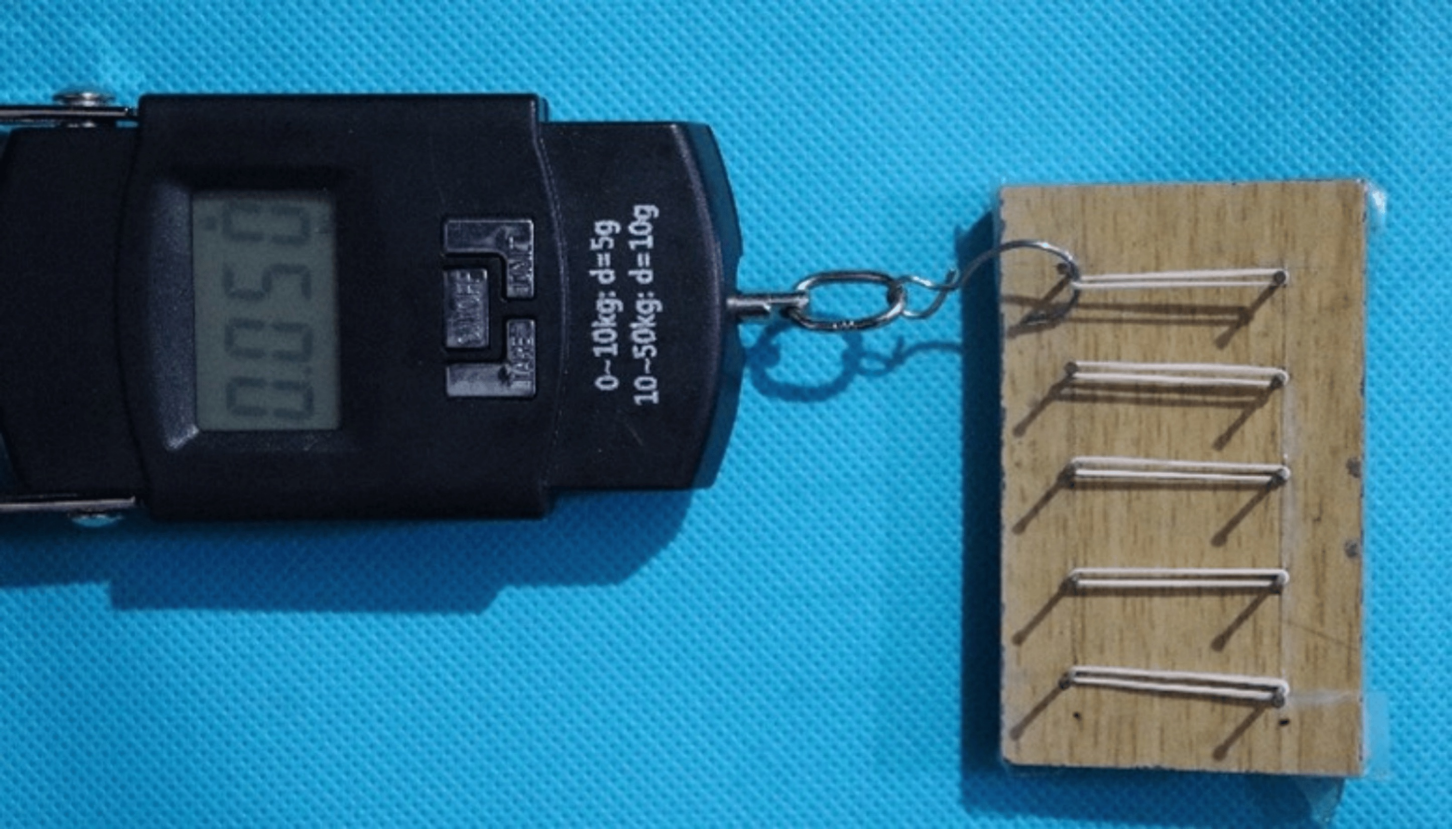
Force values recorded using digital force gauge

Force values are evaluated using a digital force gauge and recorded (Figure 4).

Force decay was determined using the following formula [5].

$\text{Force decay (\%)} = \left( \frac{F - f}{F} \right) \times 100$

Where F is the initial force before engagement of samples on custom jigs, and f is the force measurement at a specified time interval (one, three, 12, and 24 hours).

**Figure 5:**
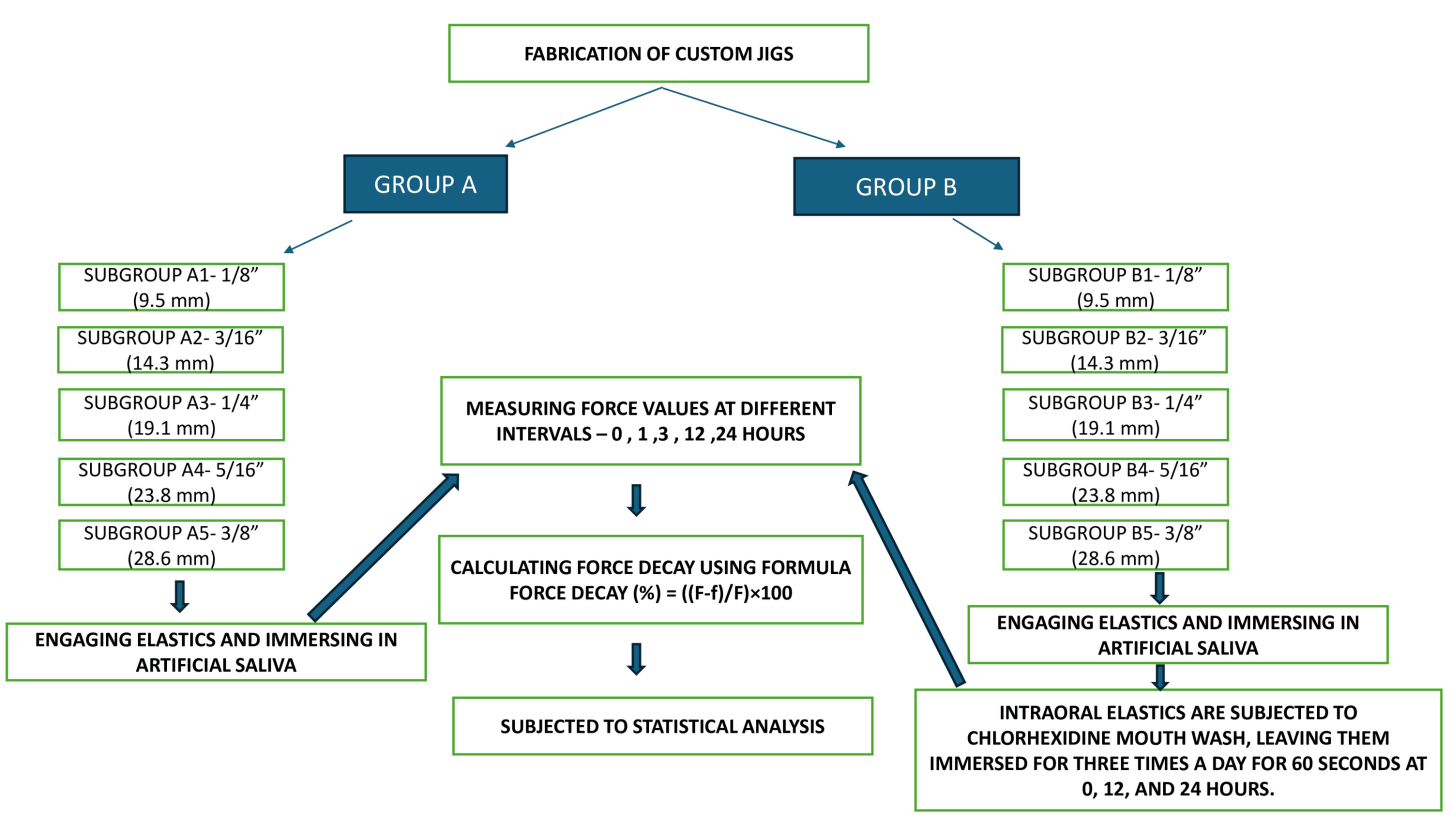
Methodological flowchart

The data is expressed as means and standard deviations for both groups. Comparison of means between two groups is done using a t-test. Comparison of means across different time periods was performed using ANOVA. A p < 0.05 is considered to be significant. All analyses were carried out using SPSS Statistics version 26 (IBM Corp. Released 2019. IBM SPSS Statistics for Windows, Version 26.0. Armonk, NY: IBM Corp.). A methodological flowchart summarizing the study design and procedures is provided (Figure 5) to give a clear overview of the experimental workflow.

## Results

The comparison of force decay among intraoral elastics of different dimensions (A1-A5) across time intervals demonstrated significant variations, as confirmed by ANOVA analysis. At one hour, A4 and A3 elastics exhibited the least mean force decay (Table 1), while A1 and A5 showed comparatively higher values. By three hours, although A5 had the highest force decay, A3 and A4 recorded much lower values, confirming a differential pattern of force decay. At 12 hours, A2 and A5 continued to show higher mean force decay, whereas A3 and A4 registered the lowest, reflecting progressive decay in certain elastics. By 24 hours, the mean force decay was highest in A1 and A2, while A3 had the lowest. Overall, the findings indicate that force decay varies markedly with elastic dimension and time, with some elastics (A1, A2) exhibiting the most significant force decay, whereas others (A3, A4) show relatively stable force over 24 hours.

**Table 1:** Comparison of force decay among different elastics (A1-A5) across time intervals using ANOVA test ANOVA: analysis of variance, SD: standard deviation, CI: confidence interval

Time interval	Groups	Mean	SD	95% CI		F	SIG
				Lower	Upper		
1 hour	A1	6.2220	5.69793	1.296	6.220	9.677	0.000
	A2	3.7580	3.44164	3.178	19.106		
	A3	2.6780	3.68048	14.834	24.330		
	A4	1.7850	3.57000	16.432	28.092		
	A5	9.3960	1.26494	5.047	15.113		
3 hours	A1	8.2220	8.44727	0.045	5.311	11.154	0.000
	A2	11.1420	11.13342	-0.147	8.359		
	A3	4.1060	6.29851	-0.147	8.359		
	A4	3.5700	7.14000	0.622	12.666		
	A5	11.3960	4.96186	18.195	26.177		
12 hours	A1	16.6660	9.06067	-0.769	4.339	6.145	0.000
	A2	19.5820	7.54059	-1.138	8.278		
	A3	4.1060	6.29851	4.131	13.994		
	A4	9.0625	6.79356	8.732	13.558		
	A5	16.7520	8.21537	9.000	18.228		
24 hours	A1	24.6660	8.29311	8.491	10.301	2.582	0.050
	A2	22.2620	8.62552	7.635	15.157		
	A3	6.6440	8.77450	11.346	22.158		
	A4	11.1450	3.62940	11.346	22.158		
	A5	16.7520	8.21537	5.183	23.529		

The analysis of force decay among elastics B1-B5 across one-, three-, 12-, and 24-hour intervals demonstrates distinct variations in their mechanical performance over time. At one hour, B2 exhibited the highest mean force decay, followed closely by B1 (Table 2), while B3 and B4 displayed minimal force decay. By three hours, B2 and B5 showed higher mean force decay, while B4 maintained the lowest decay. However, the ANOVA results at this interval were not statistically significant, indicating that the intergroup differences were less pronounced. At 12 hours, B2 and B5 showed the highest force decay, and the ANOVA revealed a statistically significant difference among the groups. At 24 hours, B2 had the most significant mean force decay, although ANOVA results were not significant at this time point.

**Table 2:** Comparison of force decay among different elastics (B1-B5) across time intervals using ANOVA test ANOVA: analysis of variance, SD: standard deviation, CI: confidence interval

Time interval	Groups	Mean	SD	95% CI		F	SIG
				Lower	Upper		
1 hour	B1	4.2220	5.79452	0.077	8.367	2.917	0.031
	B2	5.5200	3.09509	3.306	7.734		
	B3	1.4280	3.19311	-0.856	3.712		
	B4	1.3320	2.97844	-0.799	3.463		
	B5	2.9660	4.06602	0.057	5.875		
3 hour	B1	4.2220	5.79452	0.077	8.367	0.844	0.505
	B2	8.2820	5.78759	4.142	12.422		
	B3	3.9360	3.62109	1.346	6.526		
	B4	1.3320	2.97844	-0.799	3.463		
	B5	7.3600	10.30185	-0.010	14.730		
12 hour	B1	4.2220	5.79452	0.077	8.367	3.638	0.012
	B2	20.7580	8.40125	14.748	26.768		
	B3	10.1420	9.03086	3.682	16.602		
	B4	14.3560	12.37578	5.503	23.209		
	B5	16.6100	20.22372	2.143	31.077		
24 hour	B1	10.0800	6.50662	5.425	14.735	1.277	0.293
	B2	22.1860	5.67103	18.129	26.243		
	B3	13.6140	6.21373	9.169	18.059		
	B4	14.3560	12.37578	5.503	23.209		
	B5	19.7040	20.01603	5.385	34.023		

The independent t-test analysis comparing force decay between mouthwash-exposed (A1-A5) and non-exposed elastics (B1-B5) reveals distinct patterns across the different time intervals (Table 3). At one hour, the force decay of A5-B5 showed a statistically significant difference (t=6.057, p<0.001), indicating that mouthwash exposure significantly reduced the force decay of this elastic. In contrast, other elastics (A1-B1, A2-B2, A3-B3, A4-B4) did not show significant differences. At three hours, A2-B2 exhibited significant differences (t=3.832, p<0.01), suggesting that mouthwash exposure reduced force decay, whereas the remaining elastics showed comparable performance. At 12 hours, A1-B1 demonstrated a significant difference (t=3.393, p<0.01), indicating decelerated force decay in the mouthwash-exposed elastic. At 24 hours, significant differences were observed in A1-B1, suggesting that mouthwash exposure continued to influence force decay in specific elastics over longer durations.

**Table 3:** Comparison of force decay between mouthwash-exposed elastics (A) and non-exposed elastics (B)

Time	Elastic pair	Mean (A)	SD (A)	Mean (B)	SD (B)	t-stat	p-value
1 hour	A1-B1	6.222	5.698	4.222	5.795	0.919	0.37
	A2-B2	3.758	3.442	5.520	3.095	-1.316	0.20
	A3-B3	2.678	3.680	1.428	3.193	0.716	0.48
	A4-B4	1.785	3.570	1.332	2.978	0.561	0.58
	A5-B5	9.396	1.265	2.966	4.066	6.057	0.00
3 hours	A1-B1	8.222	8.447	4.222	5.795	1.403	0.17
	A2-B2	11.142	11.133	8.282	5.788	3.832	0.00
	A3-B3	4.106	6.299	3.936	3.621	0.906	0.38
	A4-B4	3.570	7.140	1.332	2.978	0.202	0.84
	A5-B5	11.396	4.962	7.360	10.302	1.266	0.23
12 hours	A1-B1	16.666	9.061	4.222	5.795	3.393	0.00
	A2-B2	19.582	7.541	20.758	8.401	-1.745	0.00
	A3-B3	4.106	6.299	10.142	9.031	-1.025	0.31
	A4-B4	9.063	6.794	14.356	12.376	-1.231	0.23
	A5-B5	16.752	8.215	16.610	20.224	-0.326	0.75
24 hours	A1-B1	24.666	8.293	10.080	6.507	5.645	0.00
	A2-B2	22.262	8.626	22.186	5.671	0.326	0.75
	A3-B3	6.644	8.775	13.614	6.214	-3.076	0.00
	A4-B4	11.145	3.629	14.356	12.376	-2.006	0.06
	A5-B5	16.752	8.215	19.704	20.016	-0.513	0.61

## Discussion

Elastomeric chains are among the most commonly used materials in orthodontic practice for space closure and molar correction. However, a major limitation of these materials is their inherent tendency to undergo force decay under intraoral conditions. The loss of force over time can compromise tooth movement efficiency, prolong treatment duration, and affect the predictability of orthodontic outcomes.

Previous investigations by Kanchana et al. confirmed that intraoral elastics demonstrate significant force loss within the first 24 hours, with smaller-lumen elastics exhibiting an accelerated pattern of decay. The initial rapid decline in force is primarily due to water absorption, structural relaxation of polymer chains, and stress-relaxation mechanisms inherent to elastomeric materials [9].

The influence of lumen size on force decay observed in this study aligns with earlier findings by Bourauel et al., who found that elastic dimensions and cross-sectional geometry affect mechanical behavior. Elastics with smaller lumens exhibit greater internal stress concentration, leading to faster force loss than those with larger lumens [10]. This highlights the clinical need for orthodontists to select elastics not only based on nominal force but also considering lumen size and its effect on force sustainability.

Environmental factors, particularly exposure to chemical agents such as mouthwashes, further complicate elastic performance. Previous reports by Pithon et al. demonstrated repeated immersion in chlorhexidine accelerated elastic degradation [11]. Similarly, in vitro work by Menon et al. has shown that alcohol-based rinses cause greater deterioration in force than fluoride-containing or neutral solutions [12]. The study by Omidkhoda et al. suggests that even routine antiseptic mouth rinsing may compromise the longevity of elastic force delivery [13].

The magnitude of force loss observed is consistent with other in vitro studies, which reported that latex elastics lost up to 50% of their initial force within 24 hours [14]. Clinical studies further support these findings, showing that force degradation is greater in vivo because elastics are simultaneously exposed to thermal changes, enzymatic activity, and mechanical stresses [15]. These converging lines of evidence underscore the challenge of maintaining consistent orthodontic force delivery.

Unlike previous studies that arbitrarily stretched all intraoral elastics to fixed distances, the present study adopted a more biologically and clinically relevant approach. In reality, the distance between the two dental units in a patient is not constant. Therefore, in this study, each elastic was stretched to three times its original lumen size, maintaining proportionality relative to its dimensions. This method provides a more accurate simulation of the clinical scenario, as orthodontists routinely select elastics based on the measured distance between specific teeth rather than a predetermined, uniform stretch length. By standardizing on stretch ratio rather than absolute distance, the study eliminates bias from arbitrary elongation and enables a fair comparison of force decay across elastics with different lumen sizes.

In this study, elastics A1 and B1 consistently showed greater force decay than many of the other groups, though their patterns differed slightly. For A1, an appreciable amount of force degradation was evident as early as the one-hour interval (6.2), and this trend continued steadily with time. At three hours, A1 still showed a relatively high level of force loss (8.2), and by 12 hours, the degradation was further accentuated (16.6). By the 24-hour interval, A1 reached the most significant overall force decay (24.67), indicating a marked reduction in its ability to retain force over extended periods. B1 exhibited early force loss, with a mean decay of 4.22 at one hour, higher than most other elastics in its group. The rate of decay progressed moderately at three and 12 hours, remaining lower than B2 and B5. By 24 hours, B1 showed a mean decay of 10.08, indicating a notable but not the most significant long-term force reduction. Overall, both A1 and B1 were among the elastics that lost force more rapidly and to a greater extent, with A1 showing the steepest progression across time and B1 presenting a more gradual but still substantial reduction. These results suggest that elastics of these dimensions may not be ideal choices for situations requiring prolonged, stable force delivery.

Elastics A2 and B2 showed a pronounced tendency for force decay over time, with patterns indicating early and sustained reduction. For A2, the initial force loss at one hour was moderate (3.7), but by three hours it remained lower than A5 yet higher than the most stable elastics in the group (11.14). At 12 hours, A2 displayed the highest force decay (19.58), and this trend persisted through 24 hours, when it recorded 22.26, confirming that A2 experienced substantial force reduction over prolonged periods. B2 demonstrated a consistently higher rate of force decay across all measured intervals. At one hour, B2 already exhibited the highest mean force loss (5.52) among its group. This pattern persisted for three hours, with B2 showing the highest decay (8.28), although the statistical differences were less pronounced at this interval. At 12 hours, B2 again recorded the most significant force degradation (20.76), highlighting its sustained susceptibility to force loss. Even at 24 hours, B2 remained the most degraded among its counterparts (22.19), suggesting that it is particularly prone to continuous force decay. Overall, both A2 and B2 elastics are characterized by considerable early reduction in force, with B2 showing the most consistent and pronounced decay across all time intervals. Clinically, this suggests that these elastics may be less suitable for applications requiring prolonged, stable force delivery, and their use should be carefully considered based on treatment duration and desired force maintenance.

A3 demonstrated relatively stable force retention compared with other elastics. At one hour, its force decay was low, and this minimal loss persisted through three and 12 hours. Even at 24 hours, A3 showed the lowest overall decay (6.64), indicating strong long-term force stability. B3 also maintained low force loss throughout the observed periods. Initial decay at one hour was minimal, and although slight increases occurred at three and 12 hours, B3 remained among the elastics with the most consistent force. By 24 hours, its mean decay was still relatively low, reflecting reliable force preservation over time (13.61).

A4 exhibited consistently low force decay across all time intervals. At one hour, decay was minimal, and this stability persisted through three and 12 hours. By 24 hours, A4 maintained relatively low force loss (11.14), indicating good long-term retention. B4 similarly showed strong force stability. Initial decay at one hour was minimal, and subsequent measurements at three, 12, and 24 hours demonstrated consistently low force loss, making B4 one of the most reliable elastics in terms of sustained force.

A5 showed relatively greater force decay initially, with a noticeable loss at one hour (9.3). By three and 12 hours, the decay increased further, and although it was not the highest at 24 hours, A5 exhibited moderate long-term force reduction compared with more stable elastics. B5 displayed a similar pattern, with moderate early force loss at one hour and progressively greater decay at three and 12 hours. By 24 hours, B5 showed a substantial but not maximal reduction in force, indicating moderate stability over time.

At the 12- and 24-hour intervals, when elastics were actually exposed to mouthwash, they exhibited a significantly greater percentage of force decay than those not exposed. This indicates that the chemical composition of mouthwash accelerates the degradation of the elastic force over time. In contrast, elastics stored in dry or neutral conditions retained a relatively higher level of force during the same intervals. The difference between the exposed and non-exposed groups emphasizes that mouthwash exposure can adversely influence the mechanical stability of intraoral elastics. These findings highlight the potential impact of oral hygiene products on the clinical performance and longevity of orthodontic elastics.

From a clinical perspective, these results reinforce the importance of both material selection and patient education. Given that lumen size and environmental exposure significantly affect force retention, orthodontists should individualize elastic prescriptions and instruct patients on proper use. Replacing elastics at appropriate intervals remains essential to counteract inevitable force loss and ensure predictable treatment progress.

The present in vitro study does not fully replicate the complex intraoral environment, where factors such as salivary enzymes, pH fluctuations, and temperature variations may influence the rate of force decay. Only a single brand of elastics was evaluated, limiting the generalizability of the findings to other commercially available products. The elastics were tested under static conditions without cyclic loading, whereas intraoral use involves repeated stretching and relaxation during speech and mastication. Hence, future in vivo studies with larger sample sizes and inclusion of multiple brands are recommended to validate these findings under clinical conditions.

## Conclusions

The present in vitro study demonstrated that the force decay of intraoral elastics is influenced by both the lumen size and exposure to chemical agents such as mouthwash. Smaller-dimension elastics, specifically the 1/8-inch and 3/16-inch sizes, exhibited more rapid force decay than larger elastics. In contrast, elastics with larger lumen sizes, including the 1/4-inch and 5/16-inch elastics, showed better force retention over the same observation period. These findings highlight the importance of selecting appropriate elastic sizes in clinical orthodontic practice, as smaller lumen elastics may require more frequent replacement to maintain consistent orthodontic force levels. This proportional difference in force degradation emphasizes that lumen size is a critical factor in the longevity and effectiveness of elastics during orthodontic treatment.

Additionally, the study revealed that exposure to mouthwash further accelerated the decay in elastic force. At both the 12- and 24-hour intervals, elastics that were subjected to mouthwash demonstrated a significantly higher percentage of force loss compared to non-exposed elastics. This observation underscores the impact of chemical agents commonly used in oral hygiene on the mechanical performance of orthodontic elastics. Clinically, this suggests that patients who regularly use mouthwash may experience faster force degradation, particularly in smaller lumen elastics, potentially affecting the efficiency of tooth movement. Overall, these findings provide valuable insights for clinicians in selecting elastic sizes and determining the duration of wear for each elastic when exposed to and not exposed to mouthwash, to ensure optimal orthodontic outcomes.
